# Supplementary material for: Non-contact lower limb injuries in Rugby Union: A two-year pattern recognition analysis of injury risk factors
Source: PLoS One. 2024 Oct 24;19(10):e0307287. doi: 10.1371/journal.pone.0307287 (PMC11500902; doi:10.1371/journal.pone.0307287)
Supplement: S1 Table — (PDF) [file pone.0307287.s001.pdf]

Supporting Information

Table A: Daily musculoskeletal screening procedures.

Test	Location Assessed	Procedure
**Ankle dorsiflexion lunge**	Ankle dorsiflexion end range of motion	Athletes perform a weight-bearing lunge without shoes. Heel of the measured ankle remains in contact with the ground. Angle measured using iPhone Measure app (Level function) placing the iPhone on anterior middle portion of lower leg.
**Adductor squeeze**	Hip adductor strength	Athletes in supine position on level surface. Maximal pressure exerted by squeezing a sphygmomanometer (mmHg) pre-set to 20mmHg placed in-between knees in 45° of flexion.
**Hamstring squeeze**	Hamstring isometric strength	Athletes lay supine with arms across chest, hips and knees of one leg flexed at 90 with heel of placed on a sphygmomanometer cuff pre-set to 30mmHg fixed on a flat surface. Athletes with maximal force pushed heel into the sphygmomanometer without lifting glutes from the floor and held for 5 seconds, with peak pressure being noted.
