# Supplementary material for: Non-contact lower limb injuries in Rugby Union: A two-year pattern recognition analysis of injury risk factors
Source: PLoS One. 2024 Oct 24;19(10):e0307287. doi: 10.1371/journal.pone.0307287 (PMC11500902; doi:10.1371/journal.pone.0307287)
Supplement: Attachment — Submitted filename: Response to Reviewers.docx [file pone.0307287.s002.docx]

Dear editors and reviewers,

We would like to sincerely thank you for the time and effort you have taken to review our manuscript. Your comments have helped to improve the manuscript and we hope that it is now satisfactory for publication. We have responded to reviewer comments below in ***bold italics.***

Reviewer #1: The manuscript was written very well and in details. there are some points to be considered in the manuscript.

1- The ethical code number should be provided in the manuscript.

***Thank you for this suggestion – we have included the ethical code number on lines 132-134.***

2- The inclusion and exclusion criteria are not clear. Write them clearly with related references.

***We thank the reviewer for wanting clarity on our inclusion and exclusion criteria – the methods section has now been amended on lines 121-124 and 128-132. We have also included a definition of semi-professional sport to further clarify the demographic of the included participants on lines 118-121.***

3- Some sections are without references such as the experimental approach. why did you change the load weekly? please cite the references.

***Thank you for bringing this to our attention. We have now included references to support throughout our experimental approach between lines 136-162. Training load was measured daily for the athletes, mentioned between lines 164-172, and then converted into weekly Arbitrary Units, similar to previous literature investigating the relationship between training load and injury or physical adaptation. We have therefore included these references within this section and also in our discussion of Training Load methodology.***

***Reference 35 and 36:***

***Dubois R, Paillard T, McGrath D, Chamari K, Maurelli O, Polly S, et al. Changes in training load, running performance, lower body power and biochemical characteristics of back players throughout a professional Rugby Union season. J Hum Sport Exerc [Internet]. 2017 [cited 2020 Jul 9];12(1). Available from:*** [***http://hdl.handle.net/10045/66788***](http://hdl.handle.net/10045/66788)

***Cross MJ, Williams S, Trewartha G, Kemp SPT, Stokes KA. The Influence of In-Season Training Loads on Injury Risk in Professional Rugby Union. Int J Sports Physiol Perform. 2016 Apr;11(3):350–5.***

***These articles discuss common measures of training load within Rugby Union which include weekly means of load measured by session Ratings of Perceived Exertion. Further information about our training load measures can be seen through lines 164-172.***

***Reference 37:***

***Berthon P, Fellmann N, Bedu M, Beaune B, Dabonneville M, Coudert J, et al. A 5-min running field test as a measurement of maximal aerobic velocity. Eur J Appl Physiol. 1997 Feb 1;75(3):233–8.***

***To measure aerobic capacity, the above article discusses a common field test used across team sport. Further information about our aerobic capacity testing can be seen through lines 196-204.***

***Reference 38:***

***Shalfawi SAI, Enoksen E, Tønnessen E, Ingebrigtsen J. ASSESSING TEST-RETEST RELIABILITY OF THE PORTABLE BROWER SPEED TRAP II TESTING SYSTEM. 2012;7.***

***To ensure reliability of our 10m and 40m sprint measures, we utilised the Speed Trap testing system. Further information about how we measured sprint times can be seen through lines 189-194.***

***Reference 39:***

***Case MJ, Knudson DV, Downey DL. Barbell Squat Relative Strength as an Identifier for Lower Extremity Injury in Collegiate Athletes. J Strength Cond Res. 2020 May;34(5):1249–53.***

***Back Squat 1 repetition-max is considered a key measure of general lower limb strength in athletes, and has been previously linked to lower limb injury risk. Further information about how we measured their 1RM back squat can be seen through lines 176-187.***

***Reference 40:***

***Furlong LAM, Harrison AJ, Jensen RL. Measures of Strength and Jump Performance Can Predict 30-m Sprint Time in Rugby Union Players. J Strength Cond Res [Internet]. 2019 Apr 17 [cited 2021 May 28];Publish Ahead of Print. Available from:*** [***https://journals.lww.com/00124278-900000000-94840***](https://journals.lww.com/00124278-900000000-94840)

***To measure lower limb power, the counter movement jump test was utilised. Further information about how we measured their lower limb power can be seen through lines 206-212.***

4- Were the experimental approach and all tests done by the same physiotherapist? and how about the analyses?

***We thank the reviewer for bringing to our attention that this is unclear in our methodology. Within our Experimental Approach (lines 136-162) we have specified the role of each member of the multidisciplinary team in the data collection process. We have also mentioned that the research team were responsible for the analysis of the data on line 252.***

5- What kind of load did you use in this project?

***We thank the reviewer for this question. Details about the training load measures used can be seen through lines 164-172. We utilised the session Rating of Perceived Exertion method with the 10-point category ratio scale (CR-10), as it is an accessible and reliable measure of training load. In response to this comment, we have also included the types of training that the athletes would perform as part of their schedule on lines 168-169 for clarity.***

6- How did you measure the strength of the hamstring and adductors?

***We thank the reviewer for this question. Detailed methods used for out musculoskeletal screening tests can be found in the Supplementary Table (S1 Table) noted on line 893. We ensured our methods of measuring hamstring and adductor strength via the use of sphygmomanometers were accessible for semi-professional sport, and also proven to be valid and reliable measures when compared with handheld dynamometry for isometric adductor strength (Toohey et al., 2015: Reference 43) and isokinetic dynamometry for hamstring strength (Mondin et al., 2018: Reference 45).***

7- The results for ROM were not provided and discussed in the manuscript. please consider them.

***We thank the reviewer for bringing this to our attention. In relation to discussing the ankle dorsiflexion ROM, details of our findings relating to non-contact ankle injury predictors of preseason ankle dorsiflexion and reductions in early season dorsiflexion can be found on lines 363-364. To clarify, a preseason ankle dorsiflexion of 32.45 degrees was found to be a predictive factor, as well as a reduction of 5.05 degrees in that early season phase. For severe non-contact injuries, as seen on line 339, a reduction of -1.13 degrees in early season ankle dorsiflexion ROM was found to be a predictor of this injury type. We discuss these findings relating to ankle dorsiflexion ROM and injury risk through lines 413-415, and 453-455. This comment did make us consider other measures of lower limb ROM we could have implemented as part of our athlete monitoring regime, for example hamstring ROM, however there is conflicting evidence surrounding its role in hamstring injury propensity:***

***Sanz A, Pablos C, Ballester R, Sánchez-Alarcos JV, Huertas F. Range of motion and injury occurrence in elite spanish soccer academies. Not only a hamstring shortening—related problem. The Journal of Strength & Conditioning Research. 2020 Jul 1;34(7):1924-32.***

***Opar DA, Williams MD, Shield AJ. Hamstring strain injuries: factors that lead to injury and re-injury. Sports medicine. 2012 Mar;42:209-26.***

***And from the literature, there is a suggestion that there seems to be an “optimal” lower-limb ROM depending on the key performance indicators for each sport:***

***Brazier J, Maloney S, Bishop C, Read PJ, Turner AN. Lower extremity stiffness: considerations for testing, performance enhancement, and injury risk. The Journal of Strength & Conditioning Research. 2019 Apr 1;33(4):1156-66.***

***We have therefore included a reference to ROM in our Strengths and Limitations section for future research to consider (lines 514-517).***
